# Adapting the Emotional Dysregulation Questionnaire for South Africa: methods of adaptation and psychometric properties of the South African Emotional Dysregulation Scale

**DOI:** 10.1016/j.ssmmh.2026.100611

**Published:** 2026-06

**Authors:** Rachel Jewkes, Esnat Chirwa, Samantha Willan, Gugulethu Gigaba, Naeemah Abrahams, Leane Ramsoomar, Pinky Mahlangu, Mercilene Machisa, Charntel Paile, Jani Nöthling

**Affiliations:** 1 Gender and Health Research Unit, South African Medical Research Council, Pretoria, South Africa; 2 Office of the Executive Scientist, South African Medical Research Council, Pretoria, South Africa; 3 School of Public Health, Faculty of Health Sciences, University of the Witwatersrand, Johannesburg, South Africa; 4 The School of Applied Human Sciences (Psychology), University of KwaZulu-Natal, Durban, South Africa; 5 Faculty of Medicine and Health Sciences, Department of Psychiatry, University of Stellenbosch, Stellenbosch, South Africa; 6 School of Public Health: Faculty of Health Sciences, University of Cape Town, Cape Town, South Africa; 7 School of Health Systems and Public Health, University of Pretoria

**Keywords:** Emotional dysregulation, violence against women, intimate partner violence, upset, psychometric properties, scale adaptation, South Africa

## Abstract

**Purpose:**

Emotional dysregulation is an important aspect of psychopathology, especially borderline personality disorder, and is prevalent in populations exposed to multiple traumas, like South Africa. We describe adaptation of the emotional dysregulation questionnaire (EDQ) for South Africa and its psychometric performance.

**Methods:**

We enrolled 58 ethnically-diverse women, who had experienced severe intimate partner violence, in eight group discussions held in three Provinces. They were asked what they did when they were upset, and related questions. During the groups, cognitive interviews focused on the EDQ. We adapted the EDQ using these findings, removing very similar or poorly understood items, and drafted new items for matters raised by the women. The resulting 23 items were tested with 236 women in a pilot study.

**Results:**

Women described a range of behaviours when they were upset that indicated loss of control such as drinking heavily, being abusive, breaking things and beating their children. In the pilot, two items were highly skewed and three, measuring restricted emotional expression, were also poorly correlated with the rest of the scale and performance was poorer with them included. The remaining 18 items performed well with Cronbach’s alpha=0.862, acceptable item-rest correlations, and KMO = 0.865 (all items were above 0.80). On confirmatory factor analysis, the 18-item measure had the best fit. The measure was positively associated with depression, borderline personality disorder, PTSD and complex PTSD.

**Conclusion:**

The adapted emotional dysregulation questionnaire had robust psychometric properties and is positioned to be a useful scale with traumatised populations in South Africa.

## Introduction

Adaptive emotional regulation is generally defined as the ability to manage emotions and respond, or delay response, in a manner that is socially and contextually appropriate (Thompson, 2019). In contrast, emotional dysregulation is a key underlying feature of psychopathology (Carmassi et al., 2022; D’Agostino et al., 2017; Thompson, 2019). It has multiple indicators, which present a challenge for measurement in research. These include poor emotional awareness (Salgado et al., 2020), inadequate emotional reactivity (under or overreacting) (Förster et al., 2022), intense emotional (negative or positive) experiences and expression of emotion, negative appraisal of emotions (D’Aurizio et al., 2023; Daros & Williams, 2019), emotional rigidity (Morris & Mansell, 2018), difficulty distinguishing between one’s own emotions and those of others (Daros & Williams, 2019), inappropriate emotional responses, and difficulty attributing accurate meaning to the emotions and behaviors of others (D’Agostino et al., 2017).

Emotional dysregulation often results in erratic behaviors and unstable relationships (D’Aurizio et al., 2023). It is a core feature of borderline personality disorder (Fitzpatrick et al., 2023). It is also associated with common mental disorders such as depression, anxiety and PTSD (Daros et al., 2021). In depression and anxiety, emotional dysregulation may manifest as a reduced ability to regulate negative emotions and to employ strategies to escape negative thoughts, feelings and mood (Daros et al., 2021). In PTSD, emotional dysregulation most commonly manifests as cognitive reappraisal difficulties where trauma cues trigger intense fear responses long after the threat or initial stressor has been removed (McLean & Foa, 2017). Additionally, in complex PTSD, there may be similar features of emotional dysregulation to borderline personality disorder, but it is more often accompanied by over-regulation of emotions resulting in emotional numbing, withdrawal or dissociation (Jannini et al., 2025).

Currently, limited longitudinal research into emotional dysregulation, has hindered our understanding of cause and effect in its role in psychopathology (Thompson, 2019). However, it is accepted that childhood abuse is associated with a greater risk of borderline personality disorder, depression, anxiety and PTSD (Bozzatello et al., 2021; McKay et al., 2021). Studies investigating emotional dysregulation in children have found that abuse is associated with increased emotional negativity bias, hypervigilance, internalizing symptoms (e.g. sadness, worry, fear and social withdrawal) and externalizing symptoms (e.g. agitation, aggression, impulsivity and disruptive behaviour) (Kim-Spoon et al., 2013; Pollak, 2015; Thompson, 2019). These dysregulated responses often persist into adolescence and adulthood where they frequently present as overreaction or oversensitivity to provocation from peers and partners, and anticipation of aggression and misinterpretation of social cues (Kim & Cicchetti, 2010). This may result in contextually inappropriate responses, conflict and even violence (Bender et al., 2022). Emotional dysregulation is also a key driver of conflict in relationships characterized by intimate partner violence (IPV) where it often has a bi-directional effect between couples (Maloney et al., 2023).

Robust measures of emotional dysregulation are needed to advance research (Agako et al., 2022). The 36-item Difficulties in Emotion Regulation Scale (DERS) is the most intensively researched measure of emotional dysregulation (Gratz KL & Roemer, 2004). It has been used widely in different populations and specifically in research with women who have experienced sexual assault or rape (de Waal et al., 2022; Weiss et al., 2014; Zerubavel & Messman-Moore, 2013). Most of these settings are in high income countries and often research has been conducted with college students. It was noted that there was some duplication of items, and in response to this a briefer version was developed, the DERS-16 (Bjureberg et al., 2016). This retained 5 of the 6 sub-scales in the original structure, related to non-acceptance of emotional responses, difficulty engaging in goal-directed behavior, impulse control difficulties, limited access to effective emotion regulation strategies, and lack of emotional clarity. The DERS has established weaknesses, although some of these were addressed in the 16-item version. Gill et al have argued that the measure does not consider the inherent intensity of underlying emotions associated with some disorders, but rather the awareness, acceptance and understanding of emotions, where the latter may be inhibited by the disorder itself (Gill et al., 2021). There has also been concerns around similar items measuring different underlying constructs of emotional dysregulation and that the measure does not fully cover all aspects of emotional dysregulation, such as thinking about emotions, the exaggerated behavior associated with the emotion and recognizing the experience of secondary emotions as a result of the underlying primary emotion e.g. experiencing anger as a result of rejection or feeling inferior (Osborne et al., 2017).

Further Gill et al had questioned decisions about item inclusion in the DERS, with a lack of emotional awareness included when it was not clearly a sign of emotional dysregulation, and other aspects of emotional dysregulation being excluded notably maladaptive ones, spanning cognitive, behavioural and affective dimensions (e.g., difficulties in thinking clearly when emotional, getting drunk when distressed or becoming angry) (Gill et al., 2021). The Emotional Dysregulation Questionnaire (EDQ) was then developed to overcome some of the short comings of the DERS and to more comprehensively assess emotional dysregulation under eight domains, including; detrimental attitudes to emotions, low emotional attunement, low emotional control, reduced attentional capacity, avoidance, externalizing and internalising and reduced behavioral control (Gill et al., 2021).

The EDQ sought to address some of the limitations of the DERS by extending the items and underlying domains of emotional dysregulation to more comprehensively represent the different forms of emotional dysregulations related to different disorders (Gill et al., 2021). When comparing the predictive validity of the DERS and the EDQ in a range of disorders, including autism spectrum disorder, attention deficit hyperactivity disorder, social anxiety, obsessive-compulsive disorder, eating disorders, narcissism, psychopathy, depression, anxiety, stress, borderline personality disorders, sleeping difficulties and trait aggression, the EDQ slightly outperformed the DERS (Gill et al., 2021). The 40-item EDQ, however, was longer than the original DERS, and more complex to use as it more expressly used the sub-scales with sub-scale scoring and weighting (Gill et al., 2021). It also had items that are similar or overlapping in content, and questions remained about which emotional reactions may be contextually and culturally appropriate and under which circumstances or in which populations.

The aim of this study was to adapt the EDQ for use with South African women with exposure to severe intimate partner violence. To address this question, we qualitatively tested understanding and preference for similar questions, and asked about local behavioral responses to underlying emotions, with women experiencing severe intimate partner violence (IPV) in different settings in South Africa in two studies. We also quantitatively explored the psychometric properties of the EDQ in various adaptive forms for South African women experiencing severe IPV.

## Methods

### Qualitative 1

We held two focus group discussions (FGDs) in Durban, KwaZulu-Natal Province, as part of the pre-testing of a questionnaire that was to be used in another study(Nöthling J et al., 2025; Willan et al., 2025). Only part of the discussion addressed the question of what participants did when they were upset. All participants were women between 18 and 45 years-old and were recruited through an NGO working with women exposed to community and interpersonal violence.

One was attended by 8 participants and facilitated by a very experienced isiZulu-speaking interviewer, and the other was attended by 8 participants and facilitated by an isiZulu-speaking clinical psychologist. Both FGDs were also attended by a notetaker who recorded the FGD. They were conducted in isiZulu, and audio-recorded. The recordings and notes were then transcribed and translated into English.

The sections of the transcripts and notes on emotional dysregulation were coded thematically and the codes summarized. The emerging findings were discussed together by six project team members who drew from them suggestions on how additional questions could be framed that would capture issues emerging in the discussions.

### • Qualitative 2

Research was undertaken at three Fedisa Modikologo study sites, two are large urban areas in eThekwini, KwaZulu-Natal and Pretoria, Gauteng, and the third is a rural site in a smalltown, farming district in Stellenbosch, Western Cape during October to November 2023. The research was part of the pilot studies for the main Fediša Modikologo study (Jewkes R et al., 2025).

We established two panels of women who had experienced severe IPV and had left or remained in violent relationships at each of the three sites. One panel in Pretoria had previously been involved in other research on IPV and had been recruited through the Courts. For the other two groups, women IPV survivors were purposively recruited through GBV service providers including, NGOs offering GBV services. The panel members attended 8 sessions held weekly to discuss different concepts and cognitively test questions for the study (see the protocol paper, (Jewkes R et al., 2025)). We discussed emotional dysregulation in six panel discussions (conducted as focus groups), with a total of 42 women, held in 5 South African languages, isiZulu, isiXhosa, English, Afrikaans, and Setswana (two groups were conducted in isiZulu). Discussions were approximately 2-3 hours long, and audio recorded, and a second facilitator took notes of the discussions. The facilitators were involved in developing the semi-structured discussion guides. Facilitators also wrote observation notes after each session. The group discussions provided a lively discussion on what women did when they were upset and how that differed from stress, in addition to testing proposed questions on emotional dysregulation, in order to assess content validity, conceptual acceptability and translation. During the panel discussions the women were encouraged to discuss their own thoughts, feelings and actions and what they felt other women when upset might feel or do. The second set of discussions confirmed that we had reached a point of theoretical saturation.

The discussions were audio recorded and then translated and transcribed into English. The analysis of the open discussions was an inductive analysis, but for the discussions on the items in the EDQ, the analysis summarised the key points raised per item across the different panel discussions. Nine co-authors and other staff members who had been involved in these discussions collectively agreed the interpretation after the discussions. Transcripts and notes were further analysed by the first author who also presented the analysis to the co-authors to check interpretation.

### Quantitative Methods

The sample was recruited as a non-probability sample in four research sites in South Africa: the Western Cape, KwaZulu-Natal, Gauteng and Limpopo Provinces. Participants recruited into the pilot study were women aged 18-40 years, who identified as female at birth or subsequently self-identified, and who had a current or former male partner who subjected them to severe intimate partner violence (IPV). In the case of an ex-partner, the relationship must have ended in the 12 months preceding the interview, and the partner should still be feared, intimidating, stalking or contacting her in a way she found threatening. The operational definition of severe IPV was that she had been hit with a fist, or something that could hurt, strangled, kicked, dragged, beaten up or threatened with/injured by a weapon; or been raped or forced into sex, or engage in sexual acts against her wishes; or have had threats to kill her, threats to harm any children in order to hurt her, or stalking, and she should fear him. Alternatively, she may have experienced multiple forms of severe controlling behaviour, such as being confined to the home, prevented from working or earning an income, or experienced jealous surveillance. Further detail on the methods for the Fedisa study are described in the methods paper (Jewkes R et al., 2025).

Participants were referred to the study by formal and informal services, such as non-governmental organisations and individuals in the vicinity of the study sites who knew of women experiencing severe IPV, as well as by participant chain referral (a variation on respondent-driven sampling). On completion of an interview, participants were asked if they knew another woman in the same situation and were given a study information leaflet and up to three coupons to hand to the other woman. The analysis used baseline data from the pilot study which was conducted in April-May 2024. We enrolled 236 participants in the pilot study across the four sites.

Potential participants were screened for participation at our study sites and those eligible were enrolled and completed an interview that lasted 1.5-2 hours, with data recorded on REDCap (a computer-assisted interview programme). The questionnaire included general social and demographic questions and mental health measures (PHQ-9 for depression (Kroenke K et al., 2001), the ITQ for PTSD and c-PTSD (Cloitre M et al., 2018) and a self-administered borderline personality disorder measure(NHS 111 Wales, 2024). We tested 23 emotional dysregulation items; these are shown in the results section below. There were 16 items from the EDQ and 7 items developed after the qualitative research. The retained EDQ items mapped on to the EDQ sub-scales as follows: detrimental attitude to emotions (3 items); reduced behavioural control (3 items); avoidant (3 items); low emotional control (2 items); reduced attentional capacity (2 items); externalizing (2 items); and internalising (1 item).

### Data analysis

We analysed the qualitative data by thematically coding the notes and transcripts from the formative research. We identified codes emerging from the data and summarized the codes. We then grouped descriptions thematically and constructed an account that gave an overview of the themes, drawing out illustrative phrases in quotes.

For quantitative analysis, we used descriptive statistics (frequencies with percentages and means with standard deviations) to describe the overall sample and to show the distribution of items across the response categories. Pairwise correlations were done to explore the correlations amongst the different items in the EDQ scale and explore any clustering of items. Cronbach alpha statistic was used to assess overall internal consistency of the items. A cut-off of alpha>=0.70 was used as an indication of good internal consistency (Bland JM & Altman, 1997). Corrected item-total correlations were also used to assess how well different items fitted in with the rest of the score and was used to identify items that had low correlations and were possible candidates for removal from the scale. Exploratory Factor Analysis was done using principal axis factoring (PAF) method used to identify any subscales (latent constructs) within the 23-item EDQ scale and also assess items that do not load unto any subscale as confirmation of the item-correlation results. Factor loadings were rotated using the “oblimin” method that allows correlation between factors. We used Kaiser-Meyer-Olkin (KMO) test to assess if the data were adequate for factor analysis, using a cut-off of 0.70 as acceptable level.

Confirmatory factor analysis was done using structural equation modeling. Standardised item coefficients and variance were used to assess how well an item mapped on the EDQ scale. Model fits statistics and factor loadings (>=0.3) were used to assess the most parsimonious EDQ scale. Due to the very uneven mapping of the items to the original EDQ sub-scales, we created additive scores from the overall scales and we used simple linear regression models to assess the correlation between the EDQ scores and well-recognised psychopathologies such as borderline personality disorder, complex post-traumatic disorder (C-PTSD) as exposure variables. Adjusted R-square statistics were used to assess the strength of the linear relationship between the EDQ scale and the various psychopathologies by comparing the amount of variation explained by the exposure. All quantitative analyses were done in Stata 17(StataCorp LLC)

### Ethics

All of the research received ethical approval from the South African Medical Research Council’s Human Research Ethics Committee (EC041-10/2021, EC032-9-2023; EC032-11-2023). Women were asked to complete an informed consent form in their preferred language and informed that they could exit the group discussions or interview at any time should they wish to. Participants were given R200 (~ $10.50) for their time plus travel expenses. As needed, they received basic containment by interviewers, and further counselling and referrals, including, at times, access to shelters, or help opening a case with the police.

## Results

### 1 Qualitative research (1)

Participants clearly recognized that some people acted in particular ways when they were upset. One participant indicated that such behaviour was not considered ‘respectable’, saying that a person who behaved like that would be described as a “scumbag”. This was challenged by others in the FGD, but nonetheless reflected a strong opinion, and resonated with a perception expressed in another group in the second qualitative research, that the community labelled (or stigmatised) people who lost control over their emotions. Other participants said that someone whose behaviour changed according to their feelings would be seen as “short-tempered”, which was understood as a negative label but much milder than “scumbag”. This provided more context for understanding why a person might try to shut down feelings when upset or hide their emotions from others (both EDQ items), as clearly social norms expected and possibly implicitly rewarded such emotional control.

A reason why this might be, was shown by the fact that participants expected that a person with emotional dysregulation would behave in ways that negatively impacted others. This was something they were familiar with both in their own behaviour and that of others, and in one group they all agreed with one participant who said “most of the time it is true” when asked if people cannot control their behaviour when they are upset.

When asked ‘What kinds of things do you do when you are upset?’ women responded ‘yelling, shouting’ or that they would ‘crave for alcohol’. Others said they felt ‘fatigued, tiredness’ and would ‘sleep’. Others said they would ‘cry a lot’ or lock themselves indoors. Some participants spoke of channelling their feelings into work, saying they would ‘overwork’ or ‘clean the house and wash clothes’. One said she would ‘walk around and exercise’. Other things participants raised were that when they were upset, they made poor decisions, became reckless, swore or became abusive, threw or broke things, and drank alcohol. They also said that they might hit someone or hurt people, and that they might do something to hurt the person who hurt them. When asked “what does it mean to say ‘when I’m upset, I become out of control’? ” women said, “it meant being undisciplined and doing ‘wrong things’”.

They were also asked about whether ‘*feeling* out of control’ was different from ‘*being* out of control’. In one FGD everyone agreed that they were the same. In contrast, in another FGD there was a discussion about how they could be translated differently into isiZulu, but only one participant clearly recognized them as different in meaning.

### 2 Qualitative research (2)

In the Fedisa Modikologo qualitative study, participants were asked what they did when they were feeling upset or stressed. These were open questions and a discussion followed that was wrapped up by asking if they felt being ‘stressed’ and ‘upset’ were different. There were some contrasting differences in the way ‘stress’ and ‘upset’ were understood. One group from Durban agreed that stress was like a ‘load on you’ and was hard to express, it was ‘draining’ and could lead to physical symptoms such as neck and shoulder pain. This was similar to a group in Stellenbosch who said stress was ‘unexplainable’, but then they proceeded to explain that it was feeling ‘overloaded’, ‘feeling angry about something they do not even know’, ‘wanting to cry’ and ‘how we feel when we are under a lot of pressure’. In contrast the Durban group said ‘upset’ was externally caused, led to feeling down, could cause anger and often ‘passes quickly’. A second group in Durban struggled to explain ‘stress’, however, and just one participant suggested it meant being ‘emotionally drained’. When a person was ‘upset’, they said, they would ‘worry too much about things that they could not control, such as not being able to provide for your family’. They said ‘stress’ led to upset and stress was largely due to personal problems and ‘upset’ due to other things, such as witnessing an accident. A group in Pretoria said stress was long term and ‘blocks the mind’ whereas ‘upset’ would be when a person was in pain, hurt, or disappointed by someone, a short-term thing. This was very similar to perceptions in Stellenbosch. Participants seemed to understand stress and upset differently, with limited commonality in how the two terms were understood across, and within, the groups.

After this, they were asked to consider the 40 questions of the EDQ and for each to say if they could answer them easily, to explain what the question was asking about, to describe if the question was about things they sometimes feel, and, if so, how often they felt them. Across sites, participants felt that there was a considerable amount of repetition of questions in the EDQ and they had strong opinions about which they preferred, when they saw multiple questions asking about the same thing.

Some of the questions, they struggled to understand. For example, one group thought that ‘I have trouble controlling myself when emotional’ was very similar to ‘when I’m upset, I can’t seem to manage my emotions’, but they saw the first as a clear item and, apart from feeling it was similar, they really were not sure what the second item meant. An indication that behaviour was much better understood than emotions, women expressed the view that they understood a person may have trouble managing their behaviour when upset but struggled to understand whether that would be the same as managing their emotions. Several participants explained that they would hide their upset from some people, and be selective about who they revealed it to. Overall, in the different groups, participants struggled to differentiate between actions and emotions and often conflated them, which partly explained why they perceived so many questions as duplicates. There were strong recommendations from participants that we reduce the number of questions, and these were largely driven by perceptions of duplication.

They were then asked nine questions developed after qualitative research (1) and generally participants found them conceptually clear and resonating. It was widely agreed that, when upset, a person might make poor decisions and or become reckless. There was considerable agreement that they may swear, shout or become abusive, break or throw things, and hit someone, particularly their children. Participants widely agreed that drinking alcohol or using drugs were common when a person was upset, they added overeating (not on the original questions). Several of the participants said that when they were upset, they felt spiteful and wanted revenge. Examples given were wanting to make up stories about the person, steal their bank card, spike the person’s drink, go to a sangoma (healer) to make a spell to make the person sick and set a magic [dirt] ‘trap’ in the community to cause illness or a miscarriage, or set traps for their husband. Several mentioned that they felt they would like to get a gun and shoot their partner. These fitted well with the question about hurting the person who caused pain when you are upset. Participants were familiar with thinking about using self-harm when upset, but were not clear how common it was, and they were not strongly in agreement that a person might cut or shave their hair. A few other suggestions were raised about what they did when they were upset, including denying their partner sex, not cooking, spring cleaning, listening to music, wanting to be alone, crying, sleeping and having suicidal thoughts.

We took a lead from participants and dropped questions from the 40-item EDQ that they saw as repetitive, making selections based on their stated preference. Then we used the qualitative research to reduce the number of new items from nine to seven, dropping the less often recognised items of cutting or shaving hair and self-harm. The resulting items were tested in the pilot study survey. The items from the EDQ mapped onto its sub-scales as follows: detrimental attitude to emotions (3); reduced behavioural control (3); avoidant behaviour (3); low emotional control (2); reduced attentional capacity (2); externalizing (2); and internalising (1).

A final adaptation was in the response format. The EDQ has a 5=point Likert scoring (1 = almost never; 2 = sometimes; 3 = about half the time; 4 = most of the time; 5 = almost always). South African participants expressed preference for a simpler scoring with a 4-point scale presented as 1= strongly agree; 2=agree; 3=disagree; 4=strongly disagree. This was viewed as conceptually easier and avoided the complex mid-point assessment of ‘about half of the time’.

### 3 Quantitative research

#### 3.1 Distribution of responses to the items

The pilot survey was completed by n=243 women, with about a quarter of the women aged between 18 and 25 years (Tabke1). The interviews were conducted in six languages (SePedi, isiZulu, Afrikaans, isiXhosa, English and Setswana). Almost a third of the women held a matric certificate and a third had done further studies after leaving school. At the time of the interview, half (48.2%) were either married or cohabiting with a man and 39.1% were in a relationship but not staying together.

Table 2 shows the distribution of responses to the items. Generally, the distribution of responses showed a balance between participants that agreed or disagreed with the items. However, two items ***a*** (I find that I enjoy being sad) and ***n*** (I enjoy being angry) had a much lower mean score than the others, and this was due to relatively few participants (<10%) indicating that they ‘agreed’ or ‘strongly agreed’ with the statements. A further four items (***d***,***g***,***I & m***) were more skewed in their distribution than the others, with <30% of participants strongly disagreeing or disagreeing with the statements.

Table 3 presents pairwise correlations between items and the results show some clustering of items with strong correlations between them. The items ***d (***When I am upset, I hide my emotions from others), ***I (***When I am upset, I try to shut down the feelings ***)***, and ***m*** (It is important for me to make sure others can’t tell how I am feeling inside) seem to form a cluster that is measuring internalized/restricted emotional expression. There seem to be a clustering of items h (I have difficulty controlling myself when I am upset), l (When I am upset, I have trouble managing my behavior) and q(When I am upset, I can’t concentrate on anything), that seem to be measuring poor emotional regulation. Similarly, there is a clustering of externalized or aggressive reactions items ***r, s, t, u, v*** and ***w***.

The cluster of ***d***,***i*** and ***m*** seems not to be strongly correlated with other items in the scale. Similarly, items ***a*** (I find that I enjoy being sad) and ***n*** (I enjoy being angry) are not correlated with other items in the scale, suggesting that the items may be less connected with the emotional dysregulation measured by other items. The lack of correlation with items that had skewed distribution (a,n, d, i ,m) shown in Table 2, is also highlighted in Table 4.

#### 3.2 Internal consistency and item-rest correlations

Table 4 presents the internal consistency and item-rest correlations of the Emotional Dysregulation Questionnaire items and the seven additional items. The measures’ Cronbach’s alpha was consistently high across all versions of the scale (over 0.82). However, consistent with the pairwise correlation matrix results, the item-rest correlations for the two items ‘I find I enjoy being sad’ and ‘enjoy being angry’ (items a and n) were very low and this did not support their inclusion in the scale. Furthermore three items that asked about aspects of restricted emotional expression, ‘When I’m upset, I hide my emotions from others’, ‘When I’m upset, I try to shut down the feelings’, and ‘it is important for me to make sure others can’t tell how I am feeling inside’ (items d,i,m), had very low item-rest correlations, that were not much changed by the items being reversed. Since these are an important aspect of dysfunctional emotional expression, we considered that retaining them as a separate scale could enable further exploration of their importance, whilst recognizing that further research would be needed to see if the items worked better together in a larger sample or different study population as their Cronbach’s alpha was low (0.569). The remaining 18 items in the scale had a Cronbach’s alpha of 0.862, and all the item-rest correlations were in the acceptable range, in excess of 0.3 (0.335 was the lowest).

#### 3.3 Factor analysis

Principal factor analysis of the 23-item scale showed that all the items loaded on three factors with an Eigenvalue >1, but the predominant loading was on the first factor (Eigenvalue = 5.09) (Table 5). The three factors explain 90% of the variance in the items and 60% of the variance explained by the first factor. The overall scale had a KMO test value of 0.826, indicating overall sample adequacy for factor analysis. The second factor with Eigen value of 1.30, seemed to measure aggressive/externalized emotional expression. The factors loading on the second factor also had high loading on the dominant first factor. Items d,i,m, and n loaded on the third factor. However, items d,i, and m had higher loading, while item n had an acceptable, but negative, loading (-0.384) on this factor. Item a’s loading was negative and below the accepted level (-0.256). Items a and n had the lowest KMO test values (<0.60) and had high uniqueness values (>0.80), indicating a lack of fit with the factors. Similarly, items d,i,m, also had low KMO test values (0.570, 0.591, and 0.578 respectively) and high uniqueness value (>0.70), indicating low shared variance (low fit) with the factors extracted. Item v (when I am upset, I drink alcohol) seems to cross-load on both factors 1 and 2 and had weaker loading (0.318 and 0.316 respectively).

In a 21-item scale (having removed items a and n), items load on 2 factors with the predominant loading on the first factor (Eigen value=5.05, proportion of variance explained=66%). Overall, 83% of the variance is explained by the 2 factors and overall scale KMO test value was higher than the KMO value for the 23-item scale. All the items except for items d,i,m load on the first factor and as in the 23-item scale, the 3 items (d,i,m) have low KMO test values and high uniqueness values.

The items in the reduced 18-item scale (with d,i, and m also removed) loaded on two factors, similar to the first 2 factors extracted in the 23-item scale, 93% of the variance explained by the two factors and the first factor explaining 70% of the variance in the items. The overall KMO test value was also highest (0.865) and all individual items’ KMO test values were above 0.80. The lowest loadings (<0.40) were for items b (When I am upset, I look for someone to blame), k(When I am upset, I don’t want to change how I feel) and v (When I am upset, I drink alcohol), but these were all above 0.30. As in the 23-item scale, item on drinking alcohol seemed to cross-load on both factors.

#### 3.4 Confirmatory Factor Analysis

The same picture was found with confirmatory factor analysis (Table 6). The standardized coefficients were significant for all items except the previously problematic items a,d,i,m and n. Consistent with the uniqueness values in the factor analysis, these also had very high standardized residual variance (0.98 to 1.00). Comparing the fit statistics for initial structural models (without allowing for covariances between items), show the 23-item scale having the poorest goodness of fit statistics (CFI=0.681; TLI=0.649 ; RMSEA=0.090) and the 18-item scale having the best fit statistics (CFI=0.763; TLI=0.731 ; RMSEA=0.095). These fit statistics improved when covariances were included in the structural models e.g. the fit statistics for the 18-item scale improved by allowing covariances between the items suggested by a modification index of >0.8 (CFI=0.955; TLI=0.943; RMSEA=0.044). After allowing covariances, the18-item scale still had the best fit statistics of the 3 scales. We used a standardized coefficient of 0.30 as cut-off for poor factor loading. For all three scales (23-item, 21-item, and 18-item), covariances between items were sequentially added to the model (sequential model building) based on the Expected Parameter Change (EPC) value, starting with covariances with the highest EPC (or modification indices), while also being cognizant of not overfitting the model. Twenty item-covariances were sequentially added to the 23-item model, 18 to the 21-item model and 14 to the 18-item model. Both the exploratory and confirmatory factor analyses present evidence for an 18-item scale as the most parsimonious scale for measuring emotional dysregulation in this population.

#### 3.5 Correlations with related measures

Table 7 shows the associations between the 18-item South African Emotional Dysregulation Scale and psychological measures that would be expected to be related. This shows that the scale was positively associated with depression, borderline personality disorder, PTSD and complex PTSD.

## Discussion

The main goal of this paper was to describe the development of an adaptation of the EDQ for use in South Africa with women exposed to severe IPV and to assess the reliability and validity of the adapted version. Overall, we have shown that the adapted version has satisfactory psychometric properties and it performed better than longer versions of the measure. We have shown the face validity of the measure through testing in qualitative research in multiple samples from different parts of the country, and our recommendations to shorten the original EDQ were based on a strong similarity in viewpoints about the different items from these diverse groups. We have shown that the internal consistency of the adapted measure is excellent, the construct validity is very strong and the convergent validity was very good. We have found that a reduced set of 11 items from the original EDQ combined with the seven new items constituted a scale with robust psychometric properties that showed strong convergent validity, when the correlations with related psychological measures were examined. The results suggest that the South African adaptation of the EDQ is a valid and reliable measure of self-reported emotional dysregulation.

Emotional dysregulation is commonly referred to in psychological literature, but it has been a subject of comparatively little research on its measurement in different populations, and often is discussed without use of any standard measure (e.g. Hahn et al(Hahn et al., 2022)). The two most frequently used measures, DERS and EDQ have recognized limitations and have not been the subject of much research in South Africa. Well-recognised patterns of emotional responses to being upset span dysfunctional thoughts, feelings and behaviours, however assessing these poses challenges in South Africa. In this study we have shown that emotional vocabulary was somewhat restricted among poorly-educated South African women, and they struggled with distinguishing thoughts and feelings, and were more comfortable expressing themselves in terms of behaviours. Even though there was an indication that more extreme behaviour was stigmatised, women were very familiar with these as expressions of emotional reactions to being upset. The range of behaviours women identified as engaging in when they were upset went well beyond the items in the EDQ. Despite our two qualitative studies being conducted with different populations (rape-exposed and IPV-exposed women) and in a range of geographical sites, there were strong similarities in women’s accounts of how they reacted to being upset. The first qualitative study informed the development of an additional set of seven items for the South African EDQ, and we received a strong indication of construct validity from the second piece of qualitative research.

The currently most frequently used measures of emotional dysregulation, the 36-item and 16-item DERS and EDQ, have all been developed and tested in high income countries and there are few examples of their adaptation for and use in middle income countries, much less, low income settings, and no examples of use with women exposed to severe IPV. The settings where they have been used are quite different from South Africa, where the general population is not well educated. Furthermore, the DERS has established weaknesses, and the EDQ was developed to address these, however, its greater length and more express use of sub-scales, with sub-scale scoring and weighting, did not enhance usability(Gill et al., 2021). Mental health researchers have shown a clear appetite for a shorter measure, as shown by the development and increasing use of a 16-item DERS scale and even an 8-item version for use with an aging population(Espirito-Santo et al., 2024; Gerges et al., 2023). Our measure draws a number of valuable items from the EDQ and notably adds items that have cultural relevance to the population in our setting, and an easier response format. Whether our measure will be of use in other settings in Sub-Saharan Africa is a question that would require investigation in further research.

## Strengths and Limitations

A strength of this adaptation was that the research was conducted across different time points with different participant groups, from very diverse South African communities, and yet the findings from these different settings were very similar. We acknowledge that all the participants were women who had severe IPV exposures, but they also had exposure to multiple other traumas, including neglect and abuse in childhood, non-partner rape, and almost all had other lifetime traumatic exposure. We recognize that we cannot directly generalize from our work to the thoughts and feelings of other South African women and of South African men. Our pilot study sample of 235 was not an especially large sample, but it was adequate for testing the measure using a rule of thumb of 10 participants per scale item for a 23-item scale. Due to the limited sample size, we performed both EFA and CFA on the same sample, as splitting the dataset would have resulted in very small sample sizes for the EFA and CFA. However, our SEM model with covariances between items shows good fit to the data. Further research would be required to test the adapted measure with other populations and explore its validity in populations outside South Africa. We recognized that many of the items capture behavioural loss of control and externalising (e.g. hitting, breaking things, revenge behaviours, heavy drinking), and that in making the adaptation we have lost some of the spectrum of emotional dysregulation articulated earlier in the front end (e.g. negative appraisal). The three items measuring restricted emotionality hung together as an independent scale and we recommend their use in this way.

## Conclusions

Emotional dysregulation is an important dimension of psychopathology and, given its close inter-relationship with borderline personality disorder, is particularly prevalent in populations like South Africa’s that are widely exposed to trauma. Commonly-used measures of emotional dysregulation, the DERS and EDQ, both have weaknesses and were developed in quite different global contexts. Adaptation of the EDQ for use in a population with low levels of emotional literacy but a high level of awareness of emotional strain, such as that in South Africa, is important for mental health research in the country. We have shown that our population of severe IPV-exposed women had quite a low level of emotional awareness, acceptance and understanding and a much higher level of recognition of behaviours in response to emotional upset. As a result, they recommended adaptations to the scale to capture these aspects. We have shown that the new scale of 18-items (11 from the EDQ and 7 new ones) had robust psychometric properties and strong correlations with other measures of psychopathology. Thus, it is positioned to be a useful addition to a toolkit of culturally adapted mental health measures.

## Figures and Tables

**Table 1:** Characteristics of the sample

	Total N=243
Woman’s age group	
<=25yrs	67 (27.6%)
26-30yrs	51 (21.0%)
31-35yrs	66 (27.2%)
36-40yrs	59 (24.3%)
has completed high school	70 (28.8%)
Did further studies after leaving school	75 (30.9%)
Employment	
unemployed	175 (72.0%)
student	11 (4.5%)
manual work/domestic	29 (11.9%)
office/NGO/community	17 (7.0%)
small trader	11 (4.5%)
Earnings in past month *	
nothing	27 (11.1%)
R1-R500	42 (17.3%)
R501-R1000	59 (24.3%)
R1001-R2000	62 (25.5%)
>R2000	53 (21.8%)
Current relationship status	
married to a man	16 (6.6%)
cohabiting with a man	101 (41.6%)
boyfriend, not cohabiting	95 (39.1%)
no male partner	31 (12.8%)
Home language	
Black African languages	195 (80.2%)
Afrikaans	47 (19.3%)
English	1 (0.4%)

* $1=R19.24

**Table 2:** Distribution of responses to the items

	Items description			Strongly disagree (1)	disagree (2)	agree (3)	strongly agree (4)
		mean	sd	%	%	%	%
a	I find that I enjoy being sad	**1.75**	**0.67**	35.8	55.1	**7.0**	**2.1**
b	When I am upset, I look for someone to blame	2.05	0.81	24.7	51.0	18.9	5.3
c	I have trouble controlling myself when I am emotional	2.67	0.88	8.6	34.2	38.3	18.9
d	When I am upset, I hide my emotions from others	2.99	0.82	5.8	16.5	**51.0**	**26.7**
e	I over-exaggerate to others how upset I am	2.18	0.76	14.4	60.5	18.1	7.0
f	When I am upset, I have trouble controlling my emotions	2.55	0.78	8.2	37.9	44.4	9.5
g	When I am upset, I can’t concentrate on anything	2.88	0.76	4.5	22.2	53.9	19.3
h	I have difficulty controlling myself when I am upset	2.61	0.79	6.2	39.5	41.2	13.2
i	When I am upset, I try to shut down the feelings	2.83	0.74	4.9	22.6	**57.2**	**15.2**
j	When I am upset, I think that I am useless	2.67	0.84	7.4	34.2	41.6	16.5
k	When I am upset, I don’t want to change how I feel	2.37	0.8	11.1	49.4	30.5	9.1
l	When I am upset, I have trouble managing my behavior	2.46	0.78	7.8	48.6	33.3	10.3
m	It is important for me to make sure others can’t tell how I am feeling inside	3.00	0.77	4.5	16.5	**53.9**	**25.1**
n	I enjoy being angry	**1.77**	**0.6**	30.5	63.0	**5.3**	**1.2**
o	When I am upset, I find it hard to feel better	2.72	0.7	4.5	28.8	56.8	9.9
p	When I am upset, I can’t think about other things	2.74	0.72	3.7	30.9	52.7	12.8
q	When I am upset, I make poor decisions	2.60	0.81	7.0	40.3	38.7	14.0
r	When I am upset, I become reckless	2.40	0.79	9.9	50.2	30.5	9.5
s	When I am upset, I swear or become abusive	2.26	0.8	14.4	53.5	24.3	7.8
t	When I am upset, I break or throw things	2.16	0.74	15.6	57.6	21.8	4.9
u	When I am upset, I hit and can even hurt people	2.09	0.75	18.5	58.8	17.3	5.3
v	When I am upset, I drink alcohol	2.30	0.86	16.0	47.7	25.9	10.3
w	When I am upset, I do something to hurt the person who hurt me	2.31	0.79	11.5	54.3	25.5	8.6

**Table 3:** Item pairwise correlations

	a	b	c	d	e	f	g	h	i	j	k	l	m	n	o	P	q	r	s	t	u	v
a	1.00																					
b	0.145	1.00																				
c		0.238	1.00																			
d				1.00																		
e		0.276	0.217		1.00																	
f		0.240	0.427		0.198	1.00																
g		0.144	0.194			0.236	1.00															
h	0.131	0.295	0.465		0.335	0.489	0.374	1.00														
i				0.250			0.131		1.00													
j		0.206	0.189		0.207	0.219	0.268	0.360	0.212	1.00												
k	0.165	0.215	0.156		0.272	0.172		0.288		0.177	1.00											
l		0.324	0.380		0.377	0.518	0.306	0.622		0.406	0.357	1.00										
m				0.445					0.222				1.00									
n	0.313	0.152			0.143						0.161	0.144	-0.145	1.00								
o		0.215	0.133			0.224	0.261	0.236		0.234	0.210	0.319		0.144	1.00							
p		0.157			0.203	0.186	0.431	0.210		0.341	0.188	0.384			0.454	1.00						
q			0.353		0.236	0.372	0.248	0.424		0.415	0.144	0.461			0.221	0.281	1.00					
r		0.163	0.280		0.179	0.296	0.289	0.429		0.351	0.137	0.464		0.163	0.185	0.379	0.510	1.00				
s			0.148			0.225		0.235				0.353					0.273	0.212	1.00			
t		0.153	0.226		0.177	0.327	0.209	0.409		0.172	0.170	0.406			0.135	0.162	0.327	0.328	0.546	1.00		
u			0.278		0.137	0.356	0.149	0.319		0.128	0.195	0.410					0.353	0.339	0.462	0.626	1.00	
v			0.153		0.183	0.149		0.192		0.127	0.128	0.330			0.142	0.159	0.264	0.253	0.313	0.325	0.287	1.00
w		0.184	0.278		0.253	0.365		0.307		0.143	0.253	0.429		0.142		0.184	0.358	0.205	0.365	0.353	0.494	0.310

Blanks represent non-significant pairwise correlation at α=0.05

**Table 4:** Internal consistency and item-rest correlations

Item	Items description	Full scale (23 items)	Full scale (23 items)†	Reduced scale (21 items)†	Reduced scale (18 items)	separate 3 d,I,m items
a	I find that I enjoy being sad	0.1307	0.1458			
b	When I am upset, I look for someone to blame	0.35	0.3667	0.3503	0.3355	
c	I have trouble controlling myself when I am emotional	0.4267	0.4128	0.4288	0.4518	
d	When I am upset, I hide my emotions from others	-0.0347	0.1006	0.0884		0.4443
e	I over-exaggerate to others how upset I am	0.3767	0.3632	0.3504	0.3724	
f	When I am upset, I have trouble controlling my emotions	0.5145	0.5242	0.5349	0.5393	
g	When I am upset, I can’t concentrate on anything	0.3531	0.3233	0.3444	0.3676	
h	I have difficulty controlling myself when I am upset	0.6339	0.6224	0.6329	0.654	
i	When I am upset, I try to shut down the feelings	0.0452	-0.0023	-0.0121		0.2775
j	When I am upset, I think that I am useless	0.4322	0.3746	0.3794	0.423	
k	When I am upset, I don’t want to change how I feel	0.3625	0.3615	0.3417	0.3443	
l	When I am upset, I have trouble managing my behavior	0.7323	0.7395	0.748	0.755	
m	It is important for me to make sure others can’t tell how I am feeling inside	0.0384	0.0046	-0.0124		0.4221
n	I enjoy being angry	0.141	0.1814			
o	When I am upset, I find it hard to feel better	0.3751	0.3507	0.3443	0.3604	
p	When I am upset, I can’t think about other things	0.4376	0.392	0.4039	0.4303	
q	When I am upset, I make poor decisions	0.5572	0.5509	0.5705	0.5808	
r	When I am upset, I become reckless	0.5485	0.5306	0.5265	0.5395	
s	When I am upset, I swear or become abusive	0.3809	0.3542	0.3669	0.3876	
t	When I am upset, I break or throw things	0.5207	0.5284	0.5513	0.5452	
u	When I am upset, I hit and can even hurt people	0.5023	0.5085	0.5187	0.5252	
v	When I am upset, I drink alcohol	0.3508	0.3064	0.3181	0.359	
w	When I am upset, I do something to hurt the person who hurt me	0.4844	0.5118	0.517	0.5046	
	**Overall Cronbach alpha**	**0.8248**	**0.8214**	**0.8279**	**0.8621**	**0.569**

† :Items d,I & m were reversed

**Table 5:** Exploratory factor analysis of the 23-item, 21-item & 18-item scales showing rotated factor loadings

		23-Item scale			21-Item scale		18-Item scale	
Item	Item Description	Emotional dysregulation	Externalised expression	Restricted emotional expression	Emotional dysregulation	Restricted emotional expression	Emotional dysregulation	Externalised expression
a	I find that I enjoy being sad	0.156	-0.111	**-0.256**				
b	When I am upset, I look for someone to blame	**0.401**	-0.071	-0.214	**0.352**	0.111	**0.387**	-0.042
c	I have trouble controlling myself when I am emotional	**0.476**	0.144	0.039	**0.499**	0.043	**0.483**	0.125
d	When I am upset, I hide my emotions from others	-0.015	-0.008	**0.523**	-0.023	**0.197**		
e	I over-exaggerate to others how upset I am	**0.431**	-0.013	-0.050	**0.397**	0.133	**0.418**	0.007
f	When I am upset, I have trouble controlling my emotions	**0.558**	0.195	-0.059	**0.594**	-0.011	**0.566**	0.183
g	When I am upset, I can’t concentrate on anything	**0.472**	-0.158	0.162	**0.390**	0.342	**0.482**	-0.189
h	I have difficulty controlling myself when I am upset	**0.711**	0.102	-0.003	**0.704**	0.136	**0.715**	0.093
i	When I am upset, I try to shut down the feelings	0.084	-0.09	**0.414**	0.042	**0.261**		
j	When I am upset, I think that I am useless	**0.542**	-0.175	0.158	**0.445**	0.380	**0.533**	-0.171
k	When I am upset, I don’t want to change how I feel	**0.387**	0.006	-0.152	**0.362**	0.063	**0.371**	0.038
l	When I am upset, I have trouble managing my behavior	**0.788**	0.160	-0.073	**0.796**	0.091	**0.789**	0.156
m	It is important for me to make sure others can’t tell how I am feeling inside	0.083	-0.013	**0.521**	0.067	**0.230**		
n	I enjoy being angry	0.162	-0.068	**-0.384**				
o	When I am upset, I find it hard to feel better	**0.453**	-0.166	0.012	**0.365**	0.298	**0.448**	-0.166
p	When I am upset, I can’t think about other things	**0.550**	-0.223	0.137	**0.439**	0.428	**0.554**	-0.246
q	When I am upset, I make poor decisions	**0.605**	0.174	0.057	**0.628**	0.072	**0.615**	0.146
r	When I am upset, I become reckless	**0.602**	0.069	0.027	**0.584**	0.147	**0.598**	0.063
s	When I am upset, I swear or become abusive	0.295	**0.577**	0.055	**0.474**	-0.383	0.296	**0.574**
t	When I am upset, I break or throw things	**0.457**	**0.578**	-0.009	**0.630**	-0.360	**0.465**	**0.562**
u	When I am upset, I hit and can even hurt people	**0.435**	**0.604**	-0.024	**0.616**	-0.395	**0.437**	**0.603**
v	When I am upset, I drink alcohol	**0.318**	0.316	0.158	**0.404**	-0.108	**0.317**	0.310
w	When I am upset, I do something to hurt the person who hurt me	**0.448**	**0.413**	-0.146	**0.563**	-0.264	**0.447**	**0.418**

**Table 6:** Coefficients and residual variance from Confirmatory Factor Analysis Confirmatory Factor Analysis

		23-Item scale			21-Item scale			18-Item scale		
Item	Item Description	Coef(95%CI)	p-value	Var(95%CI)	Coef(95%CI)	p-value	Var(95%CI)	Coef(95%CI)	p-value	Var(95%CI)
a	I find that I enjoy being sad	**0.11(-0.02,0.24)**	**0.102**	**0.99(0.96,1.02)**						
b	When I am upset, I look for someone to blame	0.36(0.24,0.48)	<0.001	0.87(0.79,0.96)	0.36(0.24,0.48)	<0.001	0.87(0.79,0.96)	0.36(0.24,0.48)	<0.001	0.87(0.79,0.96)
c	I have trouble controlling myself when I am emotional	0.50(0.40,0.61)	<0.001	0.75(0.65,0.86)	0.51(0.40,0.61)	<0.001	0.74(0.65,0.85)	0.51(0.40,0.61)	<0.001	0.74(0.65,0.85)
d	When I am upset, I hide my emotions from others	**-0.02(-0.15,0.11)**	**0.758**	**1.00(0.99,1.01)**	**-0.02(-0.15,0.12)**	**0.788**	**1.00(0.99,1.00)**			
e	I over-exaggerate to others how upset I am	0.41(0.30,0.52)	<0.001	0.83(0.75,0.93)	0.41(0.29,0.52)	<0.001	0.84(0.75,0.93)	0.41(0.29,0.52)	<0.001	0.84(0.75,0.93)
f	When I am upset, I have trouble controlling my emotions	0.61(0.52,0.69)	<0.001	0.63(0.53,0.75)	0.61(0.52,0.69)	<0.001	0.63(0.53,0.75)	0.61(0.52,0.69)	<0.001	0.63(0.53,0.75)
g	When I am upset, I can’t concentrate on anything	0.40(0.28,0.51)	<0.001	0.84(0.76,0.94)	0.40(0.29,0.51)	<0.001	0.84(0.75,0.94)	0.40(0.29,0.51)	<0.001	0.84(0.75,0.94)
h	I have difficulty controlling myself when I am upset	0.72(0.65,0.79)	<0.001	0.48(0.39,0.59)	0.72(0.65,0.79)	<0.001	0.48(0.39,0.59)	0.72(0.65,0.79)	<0.001	0.48(0.39,0.59)
i	When I am upset, I try to shut down the feelings	**0.04(-0.10,0.17)**	**0.598**	**1.00(0.99,1.01)**	**0.04(-0.10,0.17)**	**0.579**	**1.00(0.99,1.01)**			
j	When I am upset, I think that I am useless	0.46(0.36,0.57)	<0.001	0.79(0.69,0.89)	0.46(0.36,0.57)	<0.001	0.79(0.69,0.89)	0.46(0.35,0.57)	<0.001	0.79(0.69,0.89)
k	When I am upset, I don’t want to change how I feel	0.37(0.26,0.49)	<0.001	0.86(0.78,0.95)	0.37(0.25,0.49)	<0.001	0.86(0.78,0.95)	0.37(0.25,0.49)	<0.001	0.86(0.78,0.95)
l	When I am upset, I have trouble managing my behavior	0.81(0.76,0.87)	<0.001	0.34(0.26,0.44)	0.81(0.76,0.87)	<0.001	0.34(0.26,0.44)	0.81(0.76,0.87)	<0.001	0.34(0.26,0.44)
m	It is important for me to make sure others can’t tell how I am feeling inside	**0.06(-0.07,0.19)**	**0.378**	**1.00(0.98,1.01)**	**0.06(-0.07,0.20)**	**0.354**	**1.00(0.98,1.01)**			
n	I enjoy being angry	**0.13(0.00,0.26)**	**0.053**	**0.98(0.95,1.02)**						
o	When I am upset, I find it hard to feel better	0.37(0.25,0.49)	<0.001	0.86(0.78,0.95)	0.37(0.25,0.48)	<0.001	0.87(0.78,0.96)	0.37(0.25,0.48)	<0.001	0.87(0.78,0.96)
p	When I am upset, I can’t think about other things	0.43(0.32,0.54)	<0.001	0.81(0.72,0.92)	0.43(0.32,0.54)	<0.001	0.81(0.72,0.92)	0.43(0.32,0.54)	<0.001	0.82(0.73,0.92)
q	When I am upset, I make poor decisions	0.62(0.53,0.71)	<0.001	0.61(0.52,0.73)	0.62(0.54,0.71)	<0.001	0.61(0.51,0.73)	0.62(0.54,0.71)	<0.001	0.61(0.51,0.73)
r	When I am upset, I become reckless	0.59(0.50,0.68)	<0.001	0.65(0.55,0.77)	0.59(0.50,0.68)	<0.001	0.65(0.56,0.77)	0.59(0.50,0.68)	<0.001	0.66(0.56,0.77)
s	When I am upset, I swear or become abusive	0.44(0.32,0.55)	<0.001	0.81(0.72,0.91)	0.44(0.33,0.55)	<0.001	0.81(0.72,0.91)	0.44(0.33,0.55)	<0.001	0.81(0.72,0.91)
t	When I am upset, I break or throw things	0.57(0.48,0.67)	<0.001	0.67(0.57,0.79)	0.58(0.48,0.67)	<0.001	0.67(0.57,0.78)	0.58(0.48,0.67)	<0.001	0.67(0.57,0.78)
u	When I am upset, I hit and can even hurt people	0.56(0.47,0.66)	<0.001	0.68(0.58,0.80)	0.57(0.47,0.66)	<0.001	0.68(0.58,0.80)	0.57(0.47,0.66)	<0.001	0.68(0.58,0.80)
v	When I am upset, I drink alcohol	0.39(0.27,0.50)	<0.001	0.85(0.76,0.94)	0.39(0.28,0.50)	<0.001	0.85(0.76,0.94)	0.39(0.27,0.50)	<0.001	0.85(0.76,0.94)
w	When I am upset, I do something to hurt the person who hurt me	0.54(0.44,0.63)	<0.001	0.71(0.61,0.83)	0.54(0.44,0.63)	<0.001	0.71(0.61,0.83)	0.54(0.44,0.64)	<0.001	0.71(0.61,0.83)

Fit statistics (without item covariances):23-item (CFI=0.681; TLI=0.649 ; RMSEA=0.090); 21-items (CFI=0.709; TLI=0.676 ; RMSEA=0.092); 18-items (CFI=0.763; TLI=0.731 ; RMSEA=0.095) Fit statistics (with item covariances):23-item (CFI=0.954; TLI=0.943; RMSEA=0.036); 21-items (CFI=0.959; TLI=0.950 ; RMSEA=0.036); 18-items (CFI=0.966; TLI=0.957 ; RMSEA=0.038) All results show standardized estimates.

**Table 7:** Regression models showing associations between the 18-item South African Emotional Dysregulation Scale and psychological variables

Independent variable	Coeff	95%CI	t-value	p-value	Adj-Rsquare
CESD score	0.20	0.13,0.27	5.44	<0.001	0.106
Borderline personality disorder score	0.87	0.68,1.06	9.14	<0.001	0.255
Complex PTSD score	0.47	0.31,0.62	6.01	<0.001	0.150
PTSD score	0.19	0.03,0.35	2.36	0.019	0.022
Internal stigma score	0.73	0.49,0.96	6.11	<0.001	0.133
External stigma score	0.36	0.20,0.52	4.51	<0.001	0.075

## Article Highlights

- The population of South Africa is exposed to multiple traumas and emotional dysregulation is common
- We asked trauma-exposed women to explore what they did when they were upset and their views on a standard measure
- We used information provided by South African trauma-exposed women to adapt and test the measure
- Our measure performed well in tests and was correlated with mental health problems in the way we expected
- We concluded that an 18-item measure of emotional dysregulation can be useful for mental health research in South Africa
